# Galectin-1 is a poor prognostic factor in patients with glioblastoma multiforme after radiotherapy

**DOI:** 10.1186/s12885-018-4025-2

**Published:** 2018-01-30

**Authors:** Shang-Yu Chou, Shao-Lun Yen, Chao-Cheng Huang, Eng-Yen Huang

**Affiliations:** 1 Departments of Radiation Oncology, Kaohsiung Chang Gung Memorial Hospital, 123 Ta-Pei Road, Niao-Song Dist, Kaohsiung City, 83301 Taiwan; 2 Department of Pathology, Kaohsiung Chang Gung Memorial Hospital, Chang Gung University College of Medicine, Hospital, 123 Ta-Pei Road, Niao-Song Dist, Kaohsiung City, 83301 Taiwan; 3 Department of Radiation Oncology, Xiamen Chang Gung Hospital, No. 123, Xiafei Rd., Haicang District, Fujian China; 4 School of Traditional Chinese Medicine, Chang Gung University College of Medicine, No. 259, Wenhua 1st Rd., Guishan Dist., Taoyuan City, Taiwan; 5 Department of Pathology, An Nan Hospital, China Medical University, No. 66, Sec.2, Changhe Road, Annan Dist, Tainan City, 709 Taiwan; 6 Department of Radiation Oncology, Kaohsiung Chang Gung Memorial Hospital, 123 Ta-Pei Road, Niao-Song Dist, Kaohsiung City, 83301 Taiwan

**Keywords:** Galectin-1, Glioblastoma multiforme, Adjuvant radiotherapy, Radioresistant marker

## Abstract

**Background:**

Galectin-1, a radioresistance marker, was found in our previous study to be a prognostic factor for cervical cancer. The aim of current study is to determine the prognostic significance of the galectin-1 expression level in patients with glioblastoma multiforme (GBM) undergoing adjuvant radiotherapy (RT).

**Methods:**

We included 45 patients with GBM who were treated with maximal safe surgical resection or biopsy alone followed by adjuvant RT of EQD2 (equivalent dose in 2-Gy fractions) > or = 60 Gy for homogeneous treatment. Paraffin-embedded tissues acquired from the Department of Pathology were analyzed using immunohistochemical staining for galectin-1 expression. The primary endpoint was overall survival (OS).

**Results:**

Patients with weak expression had a better median survival (27.9 months) than did those with strong expression (10.7 months; *p* = 0.009). We compared characteristics between weak and strong galectin-1 expression, and only the expression level of galectin-3 showed a correlation. The group with weak galectin-1 expression displayed a 3-year OS of 27.3% and a 3-year cancer-specific survival (CSS) of 27.3%; these values were only 5.9% and 7.6%, respectively, in the group with strong galectin-1 expression (*p* = 0.009 and 0.020, respectively). Cox regression was used to confirm that the expression level of galectin-1 (weak vs. strong) is a significant factor of OS (*p* = 0.020) and CSS (*p* = 0.022). Other parameters, such as the expression level of galectin-3, Eastern Cooperative Oncology Group (ECOG) performance, gender, surgical method, age ≥ 50 years, tumor size, or radiation field were not significant factors.

**Conclusion:**

The expression level of galectin-1 affects survival in patients with GBM treated with adjuvant RT. Future studies are required to analyze the effect of other factors, such as O(6)-methylguanine-DNA methyltransferase (MGMT)-promoter methylation status, in patients with weak and strong galectin-1 expression.

**Electronic supplementary material:**

The online version of this article (10.1186/s12885-018-4025-2) contains supplementary material, which is available to authorized users.

## Background

Glioblastoma multiforme (GBM) is the most common type of brain tumor, accounting for 60% of all malignant primary brain tumors in adults; GBM is also the most malignant type. Although GBM is rare, with an incidence of 2–3 cases per 100,000 patients with primary malignant brain tumors in Europe and North America [1], a significant increase in the incidence of GBM has been observed [2]. In general, the disease progresses rapidly and has a poor outcome. Without treatment, overall survival (OS) in GBM patients is only 3–5 months [3], and despite multimodal aggressive treatment, the median survival of GBM is still only 12 months. Currently, maximal safe surgical resection followed by adjuvant local radiotherapy (RT) and temozolomide (TMZ) is considered the standard treatment for GBM. In 2005, a large clinical trial of 575 participants randomized to treatment with standard RT vs. RT plus TMZ chemotherapy reported that the latter group survived a median of 14.6 months, as opposed to 12.1 months for the former group [4]. Nonetheless, the effectiveness of TMZ is weak in patients without O(6)-methylguanine-DNA methyltransferase (MGMT) promoter methylation [5]. Another possible approach is to target other molecules that can combine with radiation sensitizers to increase the therapeutic effect.

Galectin-1, a lectin that binds to the galactoside moiety of glycoprotein, has been associated with GBM progression via processes of migration [6], invasion [6, 7], angiogenesis [8, 9], and immune escape [10]. We first found galectin-1 to be involved in radioresistance in cervical cancer [11, 12]. However, in contrast to cervical cancer, GBM exhibits high galectin-1 expression [13, 14] and is radioresistant [15, 16]. Therefore, in this study, we sought to investigate role of galectin-1 in determining the outcomes of patients undergoing RT alone without planned concomitant nor adjuvant chemotherapy. The results confirmed our hypothesis that GBM patients with strong galectin-1 expression have poor OS.

## Methods

### Patients’ characteristics

This study was approved by the Institutional Review Board of Chang Gung Memorial Hospital (102-5087B). A total of 63 newly diagnosed GBM patients who were treated at our institution before January 1, 2002, were retrospectively evaluated. Different radiation doses may affect prognosis in GBM patients; Bleehen et al. reported that a survival advantage for patients with grades 3 and 4 astrocytoma was maintained with the high dose of 60 Gy [17]. The current standard dose for GBM at our institution is also 60 Gy, which is according to NCCN guidelines. Hence, we excluded patients with an equivalent dose in 2-Gy fractions (EQD2) < 60 Gy for homogeneous treatment due to poor outcome [17] to exclude the dose effect, which might mask the importance of galectin-1 for prognosis, and selected those with EQD2 ≥ 60 Gy. A total of 45 patients remained and were treated with maximal safe surgical resection or biopsy only followed by adjuvant RT of EQD2 ≥ 60 Gy for homogeneous treatment; the outcomes of these patients were analyzed (Table 1). We chose age ≥ 50 years as a cutoff based on a recursive partitioning analysis (RPA) study [18]; we chose a tumor size ≥ 5 cm as a cutoff because this size is a significant prognostic factor after RT [19]. Brain image analysis by magnetic resonance imaging (MRI) or computed tomography (CT) was performed before treatment. The tumor size was measured in the longest diameter on a T1-weighted axial contrast-enhanced image by brain MRI or axial contrast-enhanced image by brain CT. For multifocal GBM, the sum of the largest axial diameter for all lesions was used to determine the tumor size.

**Table 1:** Patient characteristics (*n* = 45)

Parameter	Gal-1 expression (weak)	Gal-1 expression (strong)	*p* value
Age (years)			0.464
< 50	5 (45.5%)	10 (29.4%)	
≥ 50	6 (54.5%)	24 (70.6%)	
Gender			0.732
Female	5 (45.5%)	13 (38.2%)	
Male	6 (54.5%)	21 (61.8%)	
ECOG			1.000
0–2	8 (72.7%)	26 (76.5%)	
3–4	3 (27.3%)	8 (23.5%)	
Tumor size			0.897
< 5 cm	3 (27.3%)	7 (20.6%)	
≥ 5 cm	2 (18.2%)	7 (20.6%)	
Unknown	6 (54.5%)	20 (58.8%)	
OP method			0.525
gross total removal	8 (72.7%)	17 (50.0%)	
subtotal removal + biopsy	3 (27.3%)	14 (41.2%)	
unknown	0 (0%)	3 (8.8%)	
RT field			0.736
Small	7 (63.6%)	19 (55.9%)	
Whole brain	4 (36.4%)	15 (44.1%)	
EQD2 (Gy)			0.987
Mean	66.36	66.38	
SEM	0.67	0.46	

*EQD2* equivalent dose in 2-Gy fractions, *OP* operation, *RT* radiotherapy

### Radiation therapy

Patients were assigned by the radiation oncologist either for whole-brain irradiation (19 patients) followed by tumor bed boost or for tumor-site-only irradiation with a small field throughout the entire RT course (26 patients). For the latter, 11 patients were treated with a field-in field (FIF) boost protocol with RT that consisted of daily fractions of 180 cGy applied to a large target volume at the brain tumor site. This was followed by the subsequent application of 70 cGy (total 250 cGy daily) to a reduced field of the tumor bed, for a total dose of 6250–6750 cGy. The remaining 15 patients received conventional RT with 180–200 cGy per day. All RT protocols were administered in five fractions across the week.

For whole-brain irradiation, the photon beams at 6 MV or 10 MV were delivered via two-dimensional RT (2DRT). For tumor-site-only irradiation, three-dimensional conformal RT (3D–CRT) was used. The gross tumor volume (GTV) was defined according to the contrast-enhancing tumor on the CT or MRI images and included the residual tumor, perifocal edema, and entire resection cavity. The GTV plus a safe margin of 2 cm was the clinical target volume (CTV). In the FIF boost protocol, the CTV + 1 cm margin was irradiated as the large target volume, and the CTV alone was irradiated as the boost target volume. For 3D–CRT, the CTV + 3 mm margin was the planning target volume (PTV) for daily setup variation. In the FIF boost protocol, the CTV + 1.3 cm margin was the PTV of the large target.

### Tissue microarray construction

Areas showing the histopathologic features of GBM were selected on archival hemolysin and eosin (H&E)-stained sections, and then representative areas of the tumor were marked on the corresponding paraffin block for tissue microarray (TMA) construction. Briefly, after the tissue cylinders were taken from the selected regions of the donor paraffin block, they were punched precisely into a recipient paraffin block using a tissue-arraying instrument. Multiple sections (1 μm thick) were cut and mounted onto microscope slides. The TMA sections were evaluated by the pathologist (SLY), who did not know the outcomes of the patients.

### Immunohistochemistry (IHC)

**Fig. 1:**
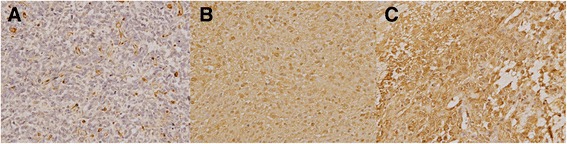
Representative cases of galectin-1 immunostaining (**a**, **b**, and **c**, respectively). **a** Tumor cells show very faint staining, but the surrounding inflammatory cells and endothelial cells shows strong staining for galectin-1. Original magnification 200X. **b** Tumor cells show moderate nuclear staining. Original magnification 200X. **c** Tumor cells show diffuse strong nuclear staining. Original magnification 200X

TMA sections were stained with an anti-galectin-1 antibody (1:40, HPA000646; rabbit to human Sigma-Aldrich, St. Louis, MO) using a DAKO REAL EnVision (DAKO, Glostrup, Denmark) and an anti-galectin-3 antibody (Santa Cruz B2C10). The immunostaining patterns for galectin-1 and -3 in the intracellular tumor portion and extracellular stromal portion of the tissue samples were recorded. IHC staining was graded as no, weak, moderate, or strong staining according to the observed intensity (Fig. 1). The staining areas were calculated by the pathologist according to the staining percentage of all tumor cells in each TMA core.

The expression level of galectin-1 can be calculated by multiplying the amount of positively stained tumor cells (%) by the staining intensity of immunoreactive tumor cells. The staining intensity of immunoreactive tumor cells was scored as follows: 0, no staining; 0.5, weak staining; 1, moderate staining; and 2, strong staining (Table 2).

**Table 2:** Staining intensity scores

Staining intensity	No staining	Weak	Moderate	Strong
Score	0	0.5	1	2

We primarily used a single grader for the immunostaining results. To dramatically reduce intraobserver differences, reference to standard slides, which revealed the reference point of the staining score, was performed before each reading. The grader was completely unaware of the outcome of the patients, and thus bias of the scoring interpretation was negligible. Normal liver tissue was employed as a negative control for galectin staining was and melanoma as a positive control.

### Follow-up and statistics

The first brain image analysis, MRI or CT, was obtained for all patients within 2–4 months after the completion of RT and then every 3–6 months or when clinically indicated, such as when new neurologic signs were observed. The optimal cutoffs of galectin-1 and galectin-3 expression for median survival were estimated by univariate analysis with the log-rank test; those for OS, cancer-specific survival (CSS) and progression-free interval were estimated by multivariate analysis with a Cox regression model. OS and CSS were measured beginning on the first day of RT. OS was measured until the date of death from any cause or the last follow-up date, and CSS was defined as a survival measure representing cancer survival until the date of death (in the absence of another cause of death). The progression-free interval indicated the time interval from the first day of RT until the date of tumor progression, as confirmed by follow-up brain CT or MRI or by death.

## Results

### Univariate and multivariate analyses of treatment outcomes

The median follow-up time was 12 months (range, 1.4–207.0 months). The 3-year OS was 11.1%, and the median survival was 12 months. Multivariate analysis using galectin expression levels as a continuous variable revealed that the expression level of galectin-1 was independent of OS (*p* = 0.046). Fourteen of the 45 patients survived for more than two years. Each patient received an EQD2 ≥ 60 Gy. Among these 14 patients, five patients received whole-brain irradiation followed by tumor bed boost and nine patients got tumor-site-only irradiation; of the latter, two were under the FIF boost protocol and seven conventional RT.

**Fig. 2:**
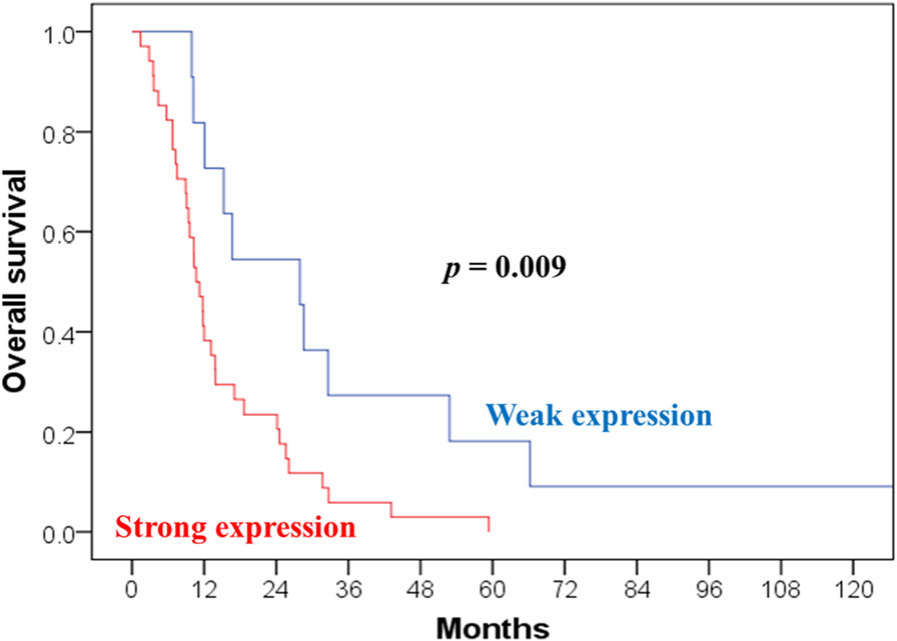
Overall survival (OS) estimated according to the expression level of galectin-1. The expression level of galectin-1 was a significant factor of OS (*p* = 0.009)

**Fig. 3:**
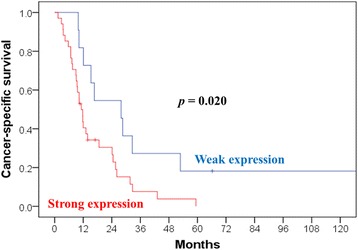
Cancer-specific survival (CSS) estimated according to the expression level of galectin-1. The expression level of galectin-1 was a significant factor of CSS (*p* = 0.020)

We attempted to determine the optimal cutoffs of galectin-1 and galectin-3 expression levels using univariate analysis (Additional file 1). Patients with weak galectin-1 expression (< 35%) had a better median survival (27.9 months) than did those with strong (≥ 35%) galectin-1 expression (10.7 months; *p* = 0.009). Among the 45 patients included, 34 showed strong galectin-1 expression and 11 weak galectin-1 expression. In addition, patients with weak galectin-3 expression (< 15%) had a better median survival (12.1 months) than did those with strong galectin-3 expression (10.7 months; *p* = 0.031). We compared characteristics between weak and strong galectin-1 expression (Table 1) and noted no significant difference. The 3-year OS was 27.3% and 5.9% (*p* = 0.009) (Fig. 2) in patients with weak and strong galectin-1 expression, respectively, and the corresponding CSS was 27.3% and 7.6%, respectively (*p* = 0.020) (Fig. 3).

We applied Cox regression to confirm the role of galectin-1 and again found it to be a significant factor of OS (*p* = 0.020; HR = 2.929; 95% CI 1.180–7.271) (Table 3). Other parameters, such as Eastern Cooperative Oncology Group (ECOG) performance, gender, surgical method, age ≥ 50 years, tumor size ≥ 5 cm, radiation field, and expression level of galectin-3, were not significant factors. CSS was also independent of galectin-1 overexpression (*p* = 0.022; HR = 2.873; 95% CI 1.163–7.101) (Table 4), as was the progression-free rate (*p* = 0.037; HR = 6.080; 95% CI 1.113–33.219) (Table 5).

**Table 3:** MVA of OS

Parameters	*p* value	HR (95% CI)
Age (≥ 50 vs. < 50 years)	0.091	1.904 (0.902–4.018)
Gender (male vs. female)	0.907	1.045 (0.501–2.179)
ECOG (3–4 vs 0–2)	0.330	1.512 (0.658–3.475)
Size (≥ 5 cm vs. < 5 cm)	0.686	0.796 (0.263–2.407)
OP method (non-gross total vs. gross total)	0.887	0.950 (0.465–1.940)
RT field (whole brain vs. small)	0.322	1.510 (0.667–3.417)
Galectin-1 (strong vs. weak)	0.020	2.929 (1.180–7.271)
Galectin-3 (strong vs. weak)	0.478	1.343 (0.594–3.034)

*MVA* multivariate statistical analysis, *OS* overall survival, *ECOG* Eastern Cooperative Oncology Group, *OP* operation, *RT* radiotherapy

**Table 4:** MVA of CSS

Parameter	*p* value	HR (95% CI)
Age (≥ 50 vs. < 50 years)	0.140	1.791 (0.826–3.883)
Gender (male vs. female)	0.822	1.092 (0.506–2.355)
ECOG (3–4 vs 0–2)	0.591	1.273 (0.527–3.075)
Size (≥ 5 cm vs. < 5 cm)	0.701	0.792 (0.241–2.603)
OP method (non-gross total vs. gross total)	0.627	0.831 (0.393–1.755)
RT field (whole brain vs. small)	0.382	1.449 (0.631–3.325)
Galectin-1 (strong vs. weak)	0.022	2.873 (1.163–7.101)
Galectin-3 (strong vs. weak)	0.993	1.004 (0.426–2.364)

*MVA* multivariate statistical analysis, *CSS* cancer-specific survival, *ECOG* Eastern Cooperative Oncology Group, *OP* operation, *RT* radiotherapy

**Table 5:** MVA of the progression-free rate

Parameter	*p* value	HR (95% CI)
Age (≥ 50 vs. < 50 years)	0.159	2.369 (0.712–7.878)
Gender (male vs. female)	0.114	0.320 (0.078–1.312)
ECOG (3–4 vs 0–2)	0.258	2.292 (0.544–9.650)
Size (≥ 5 cm vs. < 5 cm)	0.819	1.213 (0.232–6.350)
OP method (non-gross total vs. gross total)	0.023	7.408 (1.320–41.585)
RT field (whole brain vs. small)	0.609	0.716 (0.202–2.535)
Galectin-1 (strong vs. weak)	0.037	6.080 (1.113–33.219)
Galectin-3 (strong vs. weak)	0.089	0.181 (0.025–1.299)

*MVA* multivariate statistical analysis, *ECOG* Eastern Cooperative Oncology Group, *OP* operation, *RT* radiotherapy

## Discussion

Although many molecular targets have been investigated in GBM, few studies have demonstrated the relationship between molecular targets and radiosensitivity. For example, epidermal growth factor receptor (EGFR) plays a role in GBM progression [20] but has no prognostic value in these patients [21]. In addition, EGFRvIII does not affect radiosensitivity in glioblastoma cells [22]. Moreover, the EGFR inhibitor gefitinib is not a radiosensitizer for newly diagnosed GBM [23]. Ras is downstream of EGFR, and k-ras mutations frequently occur in colon cancer. Indeed, ras may play a role in the angiogenic switch in astrocytomas [24] and be involved in chemoradioresistance [25]. TLN-4601, a k-ras inhibitor [26], cannot prevent GBM recurrence [27], and bevacizumab, an anti-vascular endothelial growth factor (VEGF) antibody, was found to be ineffective for treating GBM patients undergoing RT and TMZ in two recent randomized control trials [28, 29].

Galectin-1 is expressed in human gliomas and is associated with poor differentiation [13, 14]. It also functions downstream of the EGFR pathway through H-ras by interacting and activating H-ras to form guanosine triphosphate (GTP). In addition, galectin-1 is involved cancer progression, enhances the migration and invasion of human GBM cells [6, 7] and angiogenesis [8], inhibits the anti-tumor immunity of natural killer (NK) cells [30–32], is involved in glioma chemoresistance [33], and may be considered a biomarker because serum galectin-1 levels are higher in patients with high-grade glioma than in healthy controls [10]. Despite evidence that galectin-1 may be involved in glioma progression in vitro, few animal studies on cancer progression have been performed [7, 13, 14, 31, 32], and only one human study to date reports the role of galectin-1 in glioma prognosis. In this study, Rorive et al. compared the expression level of galectin-1 in high-grade astrocytic tumors from 41 patients (26 with GBM) who survived < 12 months and > 24 months; the expression level of galectin-1 was significantly correlated with survival [13], but no details of treatment modality were reported. In the current study, galectin-1 overexpression was associated with poor survival and short time to progression following RT. To the best of our knowledge, the present study is the first report of the association of galectin-1 with poor prognosis in GBM patients following RT alone. Thus, targeting galectin-1 has potential in treatment of GBM.

In the past, the entire brain was irradiated as treatment for GBM because malignant gliomas may spread along white matter tracts [34–37]. Subsequent studies found that GBM usually recurs within 2 cm of the initial tumor volume [38, 39], and histologic analysis has shown that perifocal edema often corresponds to parenchyma infiltrated by isolated tumor cells [40]. Therefore, local irradiation, rather than whole-brain RT (WBRT), is the standard radiation treatment for GBM. Because our study was limited by early data prior to 2002, some patients had still been treated with WBRT. In GBM patients, mortality largely occurs due to local recurrence or progression in or adjacent to the resection cavity, as opposed to extracranial metastases. Although the radiation groups included in this study were inhomogeneous, we considered that the local dose is key for outcomes. Accordingly, to exclude an RT regimen effect, we used EQD2 to exclude patients who had received a local dose under 60 Gy.

Currently, the standard treatment for GBM is RT combined with TMZ [4]. Because radiation can induce galectin-1 expression in glioma cells [41], inhibition of galectin-1 expression is important for RT. Danhier et al. reported in an animal study that knockdown of galectin-1 and EGFR using nanocapsules can decrease TMZ resistance in glioblastoma [42]. TMZ also effectively knocks down galectin-1 [9, 43]. Therefore, the combination of TMZ and RT may be successful therapy.

The median survival of our GBM patients was 12.0 months, which was comparable to that in the European Organisation for Research and Treatment of Cancer - National Cancer Institute Canada (EORTC/NCIC) trial (12.1 months) [4]. In patients with and without MGMT methylation and undergoing RT alone, the median survival was 15.3 and 11.8 months [5], respectively. Although TMZ significantly improved the median survival in patients with MGMT methylation (from 15.3 to 21.7 months), this was not observed in those without MGMT methylation (from 11.8 to 12.7 months). In the present study, the median survival of patients with galectin-1 overexpression was 10.7 months, which is very similar to the median survival of patients without MGMT methylation. Despite a median survival of 27.9 months in patients with weak galectin-1 expression, the proportion of this favorable group was low, at 32.4%. Due to the lack of test reagents in our hospital, we failed to obtain the MGMT promotor methylation status, which may affect the efficacy of TMZ. In this study, all the patient received brain RT only without planned concurrent nor adjuvant TMZ. Further analysis of the effect of TMZ in patients with weak and strong galectin-1 expression is encouraged.

Galectin-3 is a common molecule studied in cancer research, especially in colon cancer, whereas only one study [44] involving an animal model has reported that galectin-3 is associated with GBM progression. Regardless, our results do not prove that galectin-3 is a prognostic factor compared with galectin-1. Galectin-1 and galectin-3 are associated with H-ras [45] and K-ras [46] signals, respectively, and H-ras [24, 47–49] but not K-ras is involved in GBM progression. Therefore, further in vitro and in vivo research is encouraged to determine the roles of galectin-1 and -3 in GBM radioresistance.

The limitations of this study are its retrospective nature and limited sample size. According to the national law of human research, informed consent for research should be obtained for work performed in our country after 2002. As we could only include patients before 2002, and the sample size was therefore small. Because these patients were treated a long time ago, almost all were dead, their charts were destroyed, and the time to progression could not be confirmed. Nonetheless, the time to progression and the survival time were correlated in patients who underwent imaging follow-up; not all patients were evaluated for time to progression, yet galectin-1 remained a significant factor of GBM progression after RT. In addition, MGMT promoter methylation is associated with patient response to alkylating agents and OS. Because the proportion of MGMT promoter methylation in the included patients was undetermined, it remains unclear whether galectin-1 expression may affect the importance of the MGMT promoter methylation status for prognosis. Future studies are required to analyze the effect of other factors, such as MGMT methylation promoter status.

## Conclusions

Ggalectin-1 expression is a poor prognostic factor for patients with GBM treated with adjuvant RT. Studies investigating the targeting of galectin-1 for radioresistance are encouraged in an effort to treat this aggressive brain tumor.

## Additional file

Additional file 1: Optimal cutoff of galectin-1 and galectin-3 for median survival (month). (DOC 38 kb)

## Abbreviations

2DRT: Two-dimensional RT
3D–CRT: Three-dimensional conformal RT
CSS: Cancer-specific survival
CTV: Clinical target volume
ECOG: Eastern Cooperative Oncology Group
EGFR: Epidermal growth factor receptor
EQD2: Equivalent dose in 2-Gy fractions
FIF: Field-in-field
GBM: Glioblastoma multiforme
GTP: Guanosine triphosphate
GTV: Gross tumor volume
MGMT: O(6)-methylguanine-DNA methyltransferase
OS: Overall survival
PTV: Planning target volume
RT: Radiotherapy
TMA: Tissue microarray
TMZ: Temozolomide
VEGF: Vascular endothelial growth factor
