# Supplementary material for: Galectin-1 is a poor prognostic factor in patients with glioblastoma multiforme after radiotherapy
Source: BMC Cancer. 2018 Jan 30;18:105. doi: 10.1186/s12885-018-4025-2 (PMC5789739; doi:10.1186/s12885-018-4025-2)
Supplement: Additional file 1: — Optimal cutoff of galectin-1 and galectin-3 for median survival (month). (DOC 38 kb) [file 12885_2018_4025_MOESM1_ESM.doc]

**Additional file 1: Table S1. Optimal cutoffs for galectin-1 and galectin-3 expression levels for assessing median survival (months**)

| Galectin levels | Weak | Strong | *p* value |
| --- | --- | --- | --- |
| Galectin-1 |  |  |  |
| 15 | 16.7 | 11.8 | 0.576 |
| 20 | 16.7 | 11.2 | 0.028 |
| 25 | 16.7 | 11.2 | 0.019 |
| 30 | 16.7 | 11.2 | 0.019 |
| 35 | 27.9 | 10.7 | 0.009 |
| 40 | 16.7 | 11.2 | 0.042 |
| 45 | 16.7 | 11.2 | 0.015 |
| 50 | 15.2 | 11.2 | 0.076 |
| Galectin-3 |  |  |  |
| 2 | 12.1 | 11.8 | 0.104 |
| 5 | 12.1 | 11.8 | 0.052 |
| 10 | 12.0 | 11.8 | 0.071 |
| 15 | 12.1 | 10.7 | 0.031 |
| 20 | 12.1 | 10.7 | 0.107 |
| 25 | 12.0 | 11.8 | 0.315 |
